# Target Screening and Single Cell Analysis of Diabetic Retinopathy and Hepatocarcinoma

**DOI:** 10.1111/jcmm.70521

**Published:** 2025-04-28

**Authors:** Yinan Shao, Bingfen Duan, Haotian Li, Xiaonan Li, Shijing Peng, Haowen Zheng, Zhipeng You

**Affiliations:** ^1^ School of Optometry, Jiangxi Medical College Nanchang University Nanchang China; ^2^ Jiangxi Provincial Institute of Ophthalmology and Vision Science Nanchang China; ^3^ Key Laboratory of Ophthalmology of Jiangxi Province Nanchang China; ^4^ Jiangxi Branch Center, National Clinical Research Center for Eye Diseases Nanchang China; ^5^ Affiliated Eye Hospital, Jiangxi Medical College Nanchang University Nanchang China; ^6^ Jiangxi Provincial Clinical Research Center for Eye Diseases Nanchang China; ^7^ The First Affiliated Hospital of Medical College Inner Mongolia University of Science and Technology Baotou China

**Keywords:** diabetic retinopathy, ferritin phagocytosis, liver cancer, Müller cells

## Abstract

The association between liver cancer and diabetes has been a longstanding focus in medical research. Current evidence suggests that diabetes is an independent risk factor for the development of liver cancer. Diabetic retinopathy (DR), a prevalent neurovascular complication of diabetes, has yet to be fully characterised concerning liver cancer. Therefore, this study seeks to identify shared genes and pathways between liver cancer and DR to uncover potential therapeutic targets. Immune infiltration and cell communication in liver cancer were analysed using the GEO single‐cell dataset GSM7494113. Single‐cell RNA sequencing data from rat retinas were obtained from the GEO datasets GSE209872 and GSE160306. Ferritin phagocytosis‐related genes were retrieved from the GeneCards database. The SeuratR package was employed for single‐cell clustering analysis, while the CellChat package assessed differences in intercellular communication. Genes shared between DR and liver cancer were identified, and the DGIDB database was consulted to predict potential drug‐gene interactions targeting membrane proteins involved in ferritin phagocytosis. Key ferritin phagocytosis (FRHG) genes were further validated using quantitative real‐time polymerase chain reaction (qRT‐PCR). After annotating the single‐cell data through dimensionality reduction and clustering, the expression of genes associated with membrane protein‐related ferritinophagy was notably elevated in both HCC and DR samples. Based on the expression of ferritinophagy‐related genes, the ferritin deposition score in Müller cells from the DR group was significantly higher than that in the control group. Cell communication analysis revealed that central hub genes associated with ferritinophagy, such as PSAP and MK, along with other signalling pathways, were significantly upregulated in the high Müller group compared to the low Müller group. In contrast, VEGF expression was enhanced in the low Müller group. Importantly, the machine learning model constructed using these key hub genes demonstrated high diagnostic efficacy for both HCC and DR. Finally, by simulating a hyperosmotic diabetic microenvironment, we confirmed in vitro that high glucose conditions significantly stimulate the expression of the shared key hub genes in both HCC and DR. The present study identified the connection between ferritinophagy‐related subgroups of cells and key hub genes in both HCC and DR, providing new insights into DR‐associated biomarkers and the shared pathological regulatory pathways with HCC. These findings further suggest potential therapeutic targets for both diseases.

## 1 Introduction

The liver plays a central role in maintaining metabolic homeostasis and detoxification processes [1]. Hepatocellular carcinoma (HCC) arises from a complex interplay of genetic predisposition and environmental factors and remains one of the most common and lethal cancers worldwide [2, 3, 4]. Chronic hyperglycemia, a hallmark of diabetes mellitus (DM), contributes to systemic metabolic dysfunction, persistent inflammation and oxidative stress, which together foster a microenvironment conducive to liver tumorigenesis. Beyond its impact on the liver, diabetes is associated with a range of microvascular complications, including DR, which may share key pathological mechanisms with HCC [5, 6, 7]. These include endothelial dysfunction, inflammatory pathways, and disturbances in iron metabolism, all contributing to vascular damage—a defining feature in both diseases.

In DR, retinal vascular damage is primarily driven by hyperglycemia‐induced oxidative stress and chronic inflammation. Similarly, in HCC, chronic liver injury results in fibrosis, inflammation and dysregulated angiogenesis, all of which play pivotal roles in disease progression [8]. Vascular endothelial growth factor (VEGF) signalling is a critical pathway in both conditions; it facilitates pathological angiogenesis in DR and supports tumour neovascularization in HCC [9]. Iron metabolism, particularly ferritinophagy, also contributes to exacerbating vascular injury in both diseases by generating ROS and amplifying inflammatory responses. Iron deposition in Müller cells of the retina and hepatocytes has been implicated in promoting cellular damage, highlighting the shared mechanisms of oxidative stress and vascular injury in DR and HCC.

Although the association between diabetes and HCC is well‐established, the molecular mechanisms that underpin this connection, particularly in the context of diabetic DR, remain insufficiently understood. The shared pathological processes between DR and HCC, including vascular dysfunction, inflammation and disturbances in iron metabolism, suggest that these diseases may not only coexist in diabetic patients but also influence each other at the molecular level. However, the molecular links between DR and HCC and how these diseases interact require further exploration. Therefore, this study aims to investigate the potential molecular connections between HCC and DR by conducting a comprehensive analysis of shared genes and signalling pathways, focusing on identifying novel therapeutic targets [9].

This study aims to deepen our understanding of the interconnected pathophysiology between DR and HCC by exploring the molecular and cellular processes underpinning both diseases. In particular, we aim to investigate how vascular damage—a hallmark of both DR and HCC—contributes to disease progression and how disturbances in iron metabolism may exacerbate these processes. By identifying overlapping pathways and biomarkers, we hope to uncover potential therapeutic targets that could be effective for both conditions, offering a more integrated approach to treating diabetic complications and liver cancer. This research will not only enhance our understanding of the shared mechanisms between DR and HCC but also provide a theoretical foundation for developing novel treatment strategies to mitigate the impact of diabetes on these devastating diseases.

## 2 Materials and Methods

### 2.1 Data Sources and Workflow

Single‐cell data were obtained from the GEO database, including the rat diabetic retinopathy dataset (GSE209872) with two control samples and three disease group samples (samples used in this study: WT: GSM6403184, DR: GSM6403184, GSM6403186, GSM6403187). In addition, scRNA‐seq data from HCC and normal tissues were downloaded from GEO datasets GSE25097, GSE281110 and GSM7494113. For bulk data, diabetic retinopathy (DR) high‐throughput sequencing data were retrieved from the GEO dataset GSE160306, which includes 20 control samples, 20 diabetic samples and 39 DR samples. The 39 DR samples were selected for this analysis.

Ferritinophagy‐related genes were identified from the GeneCards database (https://www.genecards.org/) using ‘Ferritinophagy’ as a keyword search (PMID: 37555866). After downloading the list of ferritinophagy genes, the homologate package (version 1.4.68) was used to convert human ferritinophagy genes to their rat homologues, resulting in 34 genes. Membrane protein‐related genes were obtained from the Human Proteome Database (for the gene list, see the attached). Similarly, the homologene method was applied to convert human membrane protein genes to their rat homologues. The study workflow is shown in the Graphic Abstract.

### 2.2 Single‐Cell Clustering and Annotation

The single‐cell data from GSE209872 and GSM7494113 were processed, quality‐controlled, normalised, dimensionally reduced, and clustered using the Seurat package (version 4.4.0). Initially, the CreateSeuratObject function was used to filter the single‐cell RNA sequencing data, retaining genes expressed in at least three cells and cells with more than 350 detected genes (min.cells = 3, min.features = 350). The PercentageFeatureSet function was employed to calculate mitochondrial and ribosomal gene scores based on their respective gene sets. Cells with mitochondrial and ribosomal gene score greater than 20% were excluded from further analysis. The SCTransform function was normalised (variable.features.*n* = 3000), identifying the top 3000 highly variable genes. Principal component analysis (PCA) was performed, and an inflection point plot was generated to determine the optimal number of principal components for analysis, with the first 45 principal components selected. Batch effects were corrected using Seurat's canonical correlation analysis (CCA) method. Subsequently, the FindNeighbors and FindClusters functions performed unsupervised clustering of the data post‐batch removal. The optimal resolution for clustering was determined using the clustered function, with a final resolution of 0.7, resulting in 26 clusters. Cluster visualisation was performed using UMAP. Cells were manually annotated using marker genes from the CellMarker database. The proportion of each annotated cell type was calculated, and a bar chart was generated for visualisation using ggplot2 [10].

### 2.3 Identification of Key Cells of Ferritinophagy

Based on the list of Ferritinophagy genes, the Ferritinophagy_score of each cell was calculated through ssGSEA. UMAP was used to visualise the Ferritinophagy score and analyse the difference between the disease and control groups [11, 12, 13]. Cells with different scores indicated that the cell was related to Ferritinophagy as a key Ferritinophagy cell. Then, the median value of Ferritinophagy_score was used to divide the groups with high and low Ferritinophagy ratings. After the proportion of cells in the high and low Müller groups were counted, ggplot2 was used to draw bar graphs for visualisation.

### 2.4 Cell Communication Analysis

Information exchange between cells is based on complex reactions between ligands and their receptors and activation responses of specific cell and pathway signals. The localization and study of these ligand‐receptor interactions are extremely important for understanding cell behaviour and complex biological processes. CellChat package software was used to predict the differences in cell communication between different groups with high and low Müller ratings, and the threshold was a *p* < 0.05 [14]. After different ligand‐receptor pairs were obtained, the ligand‐receptor pairs belonging to membrane proteins were selected for analysis, and the expression of ligand‐receptor pairs of membrane proteins in different cells was observed.

### 2.5 Analysis of Key Ferritinophagy Subsets

In order to analyse the heterogeneity of key Ferritinophagy cells, key Ferritinophagy cells were evaluated by secondary dimensional reduction clustering using the same method as the first clustering to obtain different key Ferritinophagy subsets. Then FindAllMarkers were used to set parameters min.pct = 0.2, only.pos = TRUE. Logic.threshold = 0.5 to search for positive marker genes of various groups. The cluster profile (version 4.1.0) package was used for functional enrichment analysis of subgroup marker genes, and key Ferritinophagy subgroups were screened according to the enrichment to autophagy pathway.

### 2.6 Protein Interaction Network and Functional Enrichment Analysis

The interaction network of key Ferritinophagy subsets of disease and control differential gene proteins was analysed using String (https://cn.string‐db.org/) database [15, 16], the confidence level was selected 0.7, and the species was selected as the rat. The correlation between key subtype characteristic genes of Ferritinophagy and Ferritinophagy score was performed using the ggcor package.

### 2.7 Gene Drugs That Potentially Target Membrane Protein‐Mediated Key Subtypes

Ferritinophagy was divided into high/low groups using ssGSEA calculated scores based on the characteristic genes of key subtypes of Ferritinophagy through bulk cohort data sets, and the differential genes between high/low groups were identified using DESeq2 (version1.42.0). The screening condition was (FDR < 0.05, |Log2FC| > 1). The protein–protein interaction (PPI) network was constructed using the String (https://cn.string‐db.org/) database, and hub gene nodes in the network were mined. Target drug analyses were performed using the DGIDB (https://dgidb.org/) database [17].

### 2.8 NCOA4, GPX4, SLC7A11 and FRHGs External Validation

In cell culture and group in the rat retina Müller cells (RMC) containing 10% fetal bovine serum (BiologicalIndustries, Israel) sugar DMEM (Solarbio, China), cultured in 5%CO_2_, 37°C incubator. The in vitro experiment was conducted with exponential‐stage RCAC‐1 cells. The influence of osmotic pressure on the experimental results was excluded. Rcac‐1 cultured in 25 mmol/L glucose medium was selected as the normal control group (CON group), RCAC‐1 cultured in 55 mmol/L glucose medium was selected as the high glucose group (HG group), and mannitol group (MA group) was selected as the control group. Model cells in the HG group were cultured with high glucose DMEM for 24 and 48 h, respectively.

#### 2.8.1 Real‐Time Quantitative Polymerase Chain Reaction (qRT‐PCR) Analysis

Total RNA was extracted from rat retina and Müller cells according to the steps using RNA extraction reagent (Servicebio, China). Using SweScriptRTIFirstStrandcDNASynthesisKit (Servicebio, China) will be a total RNA reverse transcription of cDNA. Then, RT‐PCR quantitative detection was performed using 5 × SYBRGreenqPCRMasterMix (NoneROX) (Servicebio, China). The sequence of primers used in this study is shown in the attached table. Take GAPDH as the internal parameter.

### 2.9 Cell Line Selection and Culture Conditions

Retinal pigment epithelial cells (RMC) and RCAC‐1 (rat retinal endothelial cells) were selected as model systems to investigate the mechanisms of ferroptosis, oxidative stress and immune response in DR and HCC. These cell lines were chosen based on their well‐established roles in modelling key aspects of DR and HCC. RMC cells are commonly used to study retinal endothelial dysfunction in DR, particularly in high‐glucose conditions. Their application in simulating oxidative stress, inflammation and ferroptosis in retinal cells makes them a suitable model for exploring the pathophysiology of DR. Previous studies have demonstrated that RMC cells effectively reflect the key features of retinal cell injury and iron metabolism dysregulation associated with DR [18, 19]. Similarly, RCAC‐1 cells are a validated model for studying retinal vascular damage and angiogenesis in DR and HCC. Their ability to mimic the oxidative stress and ferroptosis mechanisms involved in both diseases makes them a reliable tool for examining shared pathological pathways. The suitability of RCAC‐1 cells for modelling retinal endothelial cell responses in a high‐glucose environment has been corroborated by multiple studies [20, 21]. Both cell lines have been widely utilised in similar research, and their use in this study provides a robust platform to investigate the underlying mechanisms of ferroptosis, oxidative stress, and immune modulation in DR and HCC.

### 2.10 Statistical Analysis

All statistical analyses were done using R software (version 4.3.2, https://www.r‐project.org/). Wilcoxon rank‐sum test was used to compare the Ferritinophagy scores among different groups. When the difference *p* < 0.05, the difference was considered statistically significant.

## 3 Results

### 3.1 Interpreting the Tumour Microenvironment Heterogeneity of Liver Cancer

**FIGURE 1:**
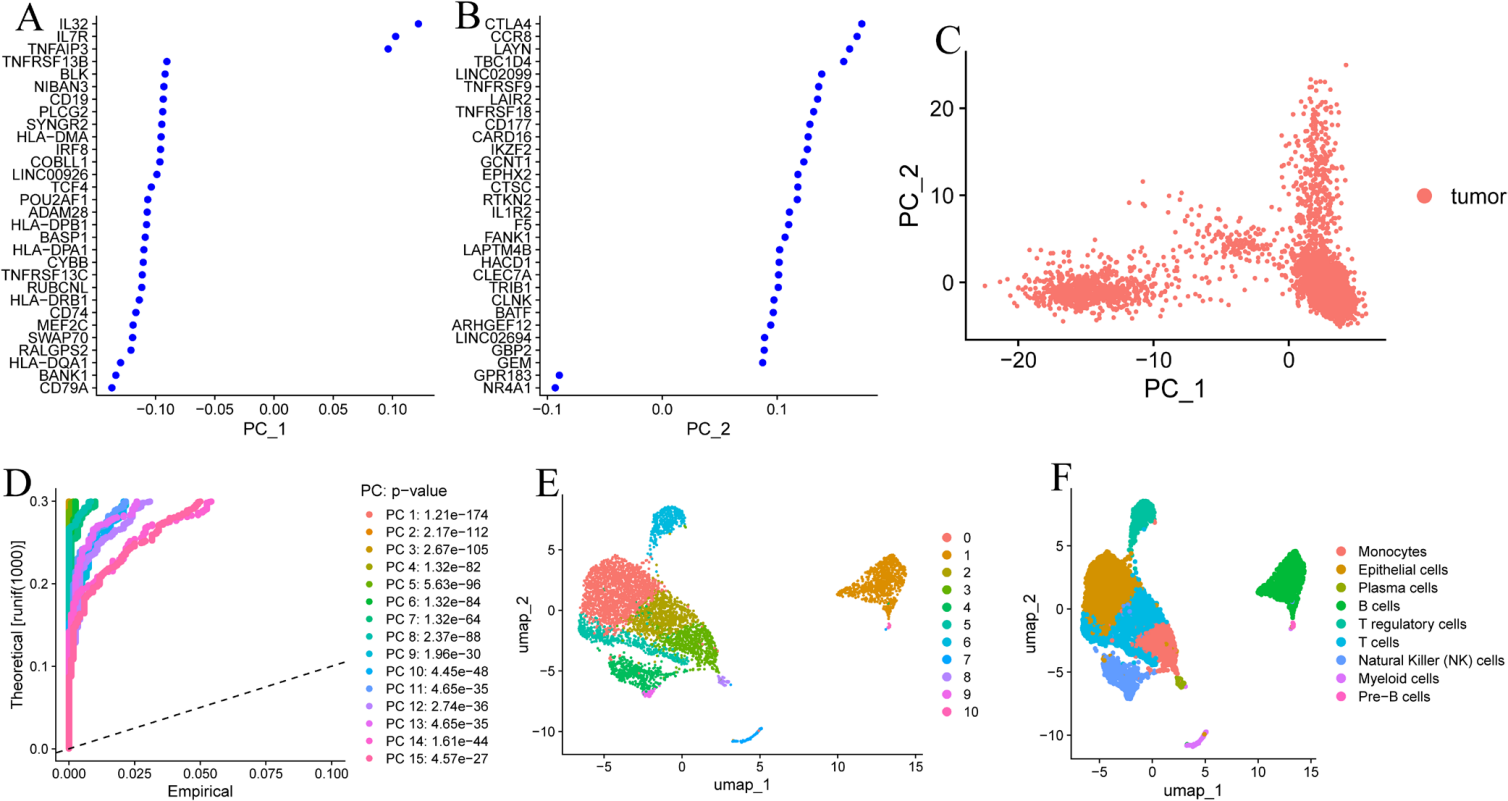
Interpreting the tumour microenvironment heterogeneity of liver cancer. (A, B) Single‐cell gene contributions of PC analysis. (C) Identification and PC analysis of identified HCC cells. (D) PC trajectory of identified sub‐cluster cells in HCC tissue. (E, F) Principle‐based and annotation‐based u‐map of HCC tissue.

First, we conducted a systematic analysis of HCC tumours and adjacent normal tissues (GSM7494113). The plot illustrates the standard deviations of PC1 and PC2 (Figure 1A–D) in a principal component analysis (PCA) to assess the variabilities among various cellular populations. Compared with other cell types, the increased dispersion of tumour cells in the PC space could mirror the previously discussed concepts regarding intricate gene regulatory networks leading to adaptive modifications. The cellular populations included in the analysis encompass epithelial cells, plasma cells, B cells, T regulatory cells, T cells, Natural Killer (NK) cells, Myeloid cells and Pre‐B cells (Figure 1E,F). This figure shows the standard deviations (A–D) of PC1 and PC2 in PCA to evaluate the variability between various cell populations. The cell populations included in the analysis of E and F include epithelial cells, plasma cells, B cells, T regulatory cells, T cells, natural killer (NK) cells, myeloid cells and pre‐B cells.

### 3.2 Uncovering TYROBP Expression and Functional Pathway in Liver Cancer

**FIGURE 2:**
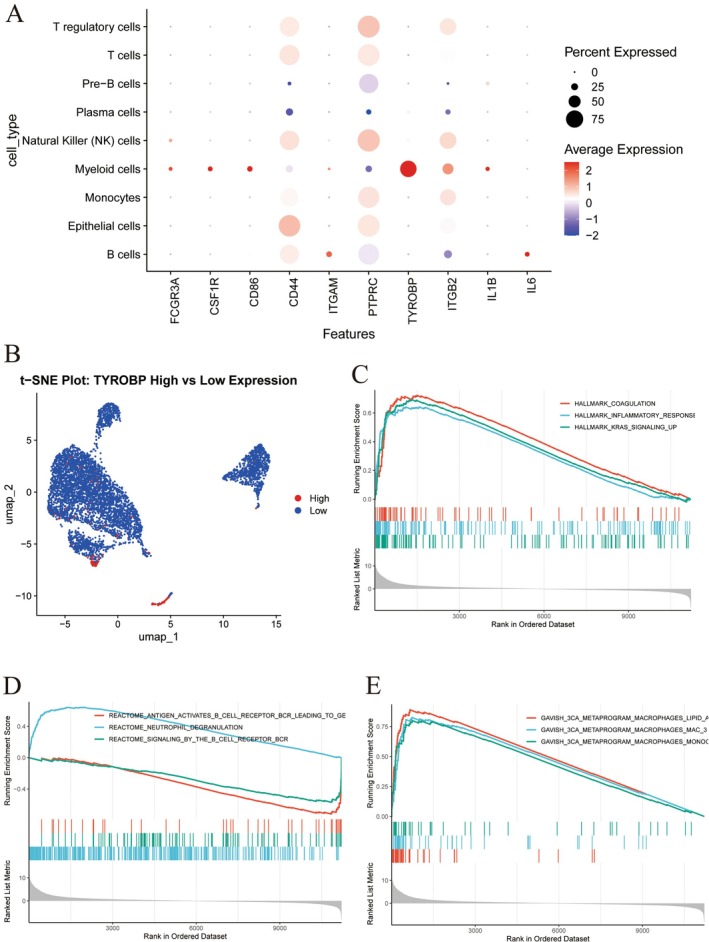
Expression and functional pathway study of TYROBP in cancer. (A) The expression of TYROB and CD44 genes in different immune cells. (B) The differential expression of TYROBP in different immune cells. (C–E) TYROBP and other gene enrichment in pathways such as marker coagulation and marker inflammatory response.

Our analysis further reveals that, compared to normal tissues, CD44 is highly expressed in multiple immune cells within HCC tissues, including T regulatory cells, T cells, NK cells, monocytes, epithelial cells and B cells. The TYROBP gene is mainly expressed in myeloid cells, with low expression levels and specificity in other cells. ITGB2 is highly expressed in NK cells and monocytes (Figure 2A), t‐NSE represents the expression of TYROBP in different immune cells (Figure 2B), and GSEA found enrichment in pathways such as hallmark‐coagulation, hallmark‐inflammatory‐response, hallmark‐kras_signalling‐up (Figure 2C–E).

### 3.3 Cell Communication in the HCC Tumour Microenvironment

**FIGURE 3:**
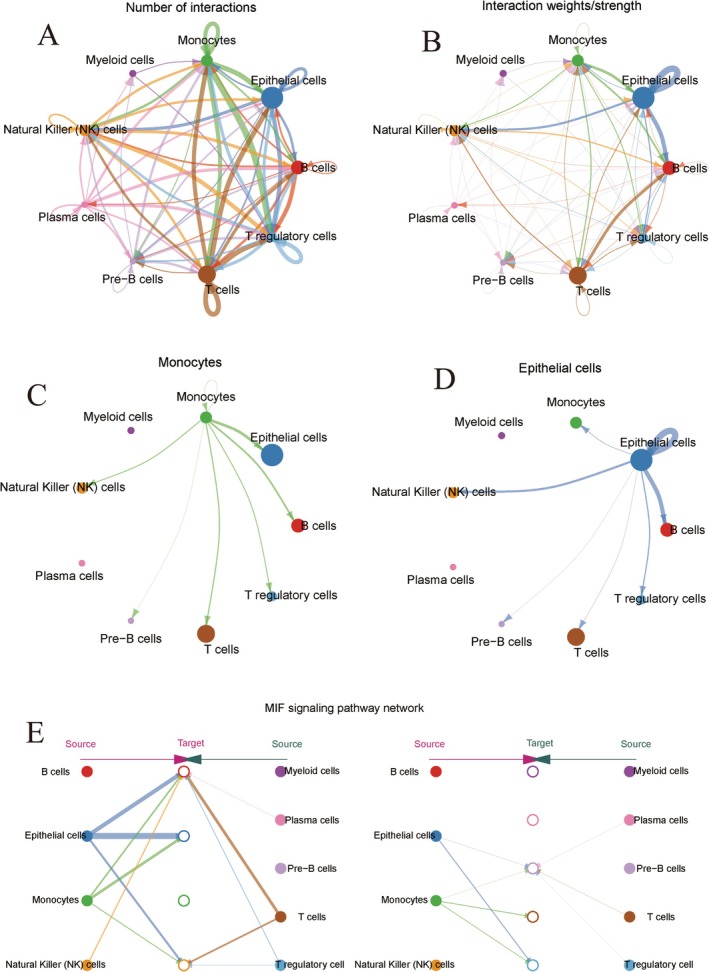
Cell communication in tumour microenvironment. (A) The number of interactions between different cell types. (B) The weights and strengths of these interactions. (C) The communication network of monocytes. (D) The communication network of endothelial cells. (E) The MIF signalling pathway network.

The interaction network between various cell types in the HCC tumour microenvironment reveals distinct patterns. T cells exhibit the highest number of interactions with tumour cells, suggesting a key role for T cells in the tumour immune response (Figure 3A). Furthermore, the strength and weight of these interactions indicate that T cells and tumour cells engage in the most robust interactions (Figure 3B). Tumour cell communication networks demonstrate substantial interactions between tumour cells and NK cells, T cells and endothelial cells, underscoring the complexity of tumour cell interactions (Figure 3C). Endothelial cells also communicate significantly with NK, T, tumour and red blood cells, with particularly strong connections to NK cells and tumour cells (Figure 3D). In the MIF signalling pathway network, endothelial cells, myeloid cells, T cells and NK cells interact extensively, with endothelial cells notably interacting with tumour cells, T cells and macrophages, highlighting their critical role in the MIF signalling pathway (Figure 3E).

### 3.4 Analysis of Cell Types and Gene Expression Dynamics

**FIGURE 4:**
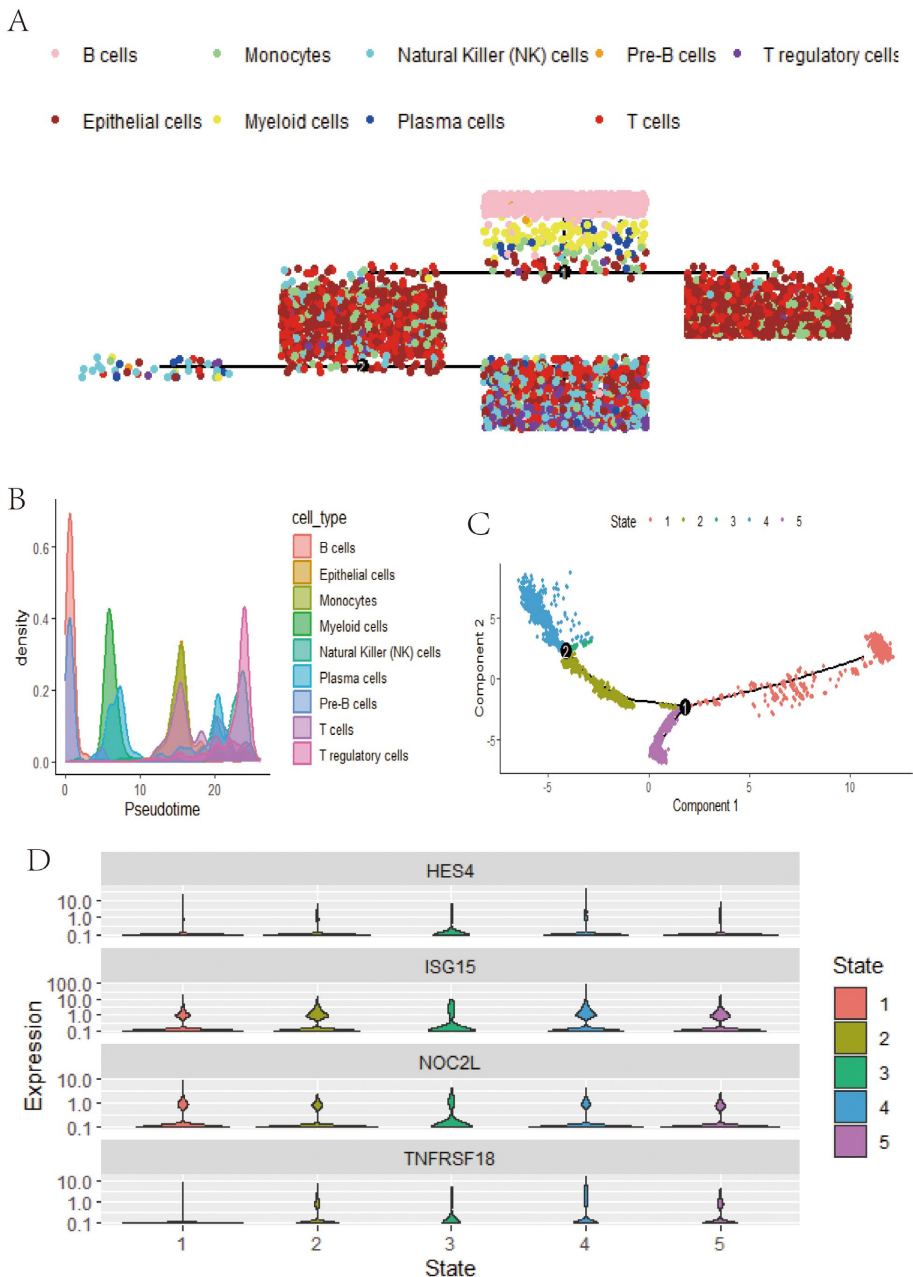
Analysis of cell types and gene expression dynamics. (A) Cell type trajectory: Different colours represent each cell type's distribution and evolutionary path along the pseudotime axis. (B) Density plot: Shows the density distribution of different cell types along the pseudotime axis, highlighting transitions in cell populations. (C) Pseudotime trajectory: A dimensionality reduction plot displays the distribution of cells in different states and their progression path. (D) Gene expression: Violin plots show the expression changes of specific genes across different cell states.

Trajectory analysis along a pseudo‐time axis reveals the differentiation paths of various cell types, with distinct cell populations such as B cells, T cells, NK cells, monocytes and others identified at different points along the axis (Figure 4A). The density distribution of these cell types along the pseudotime axis highlights specific peaks where certain cell types are more prevalent during the progression, indicating transitions and shifts in cell populations (Figure 4B). Dimensionality reduction analysis (e.g., t‐SNE or UMAP) further demonstrates the distribution of cells across different components, with colours representing distinct cell states. The trajectory path is overlaid, illustrating the progression through various cellular states (Figure 4C). Finally, violin plots reveal the expression levels of key genes (HES4, ISG15, NOC2L and TNFRSF18) across different cell states, with each state represented by a colour to show how gene expression varies during the progression (Figure 4D).

### 3.5 Gene Expression and Network Analysis

**FIGURE 5:**
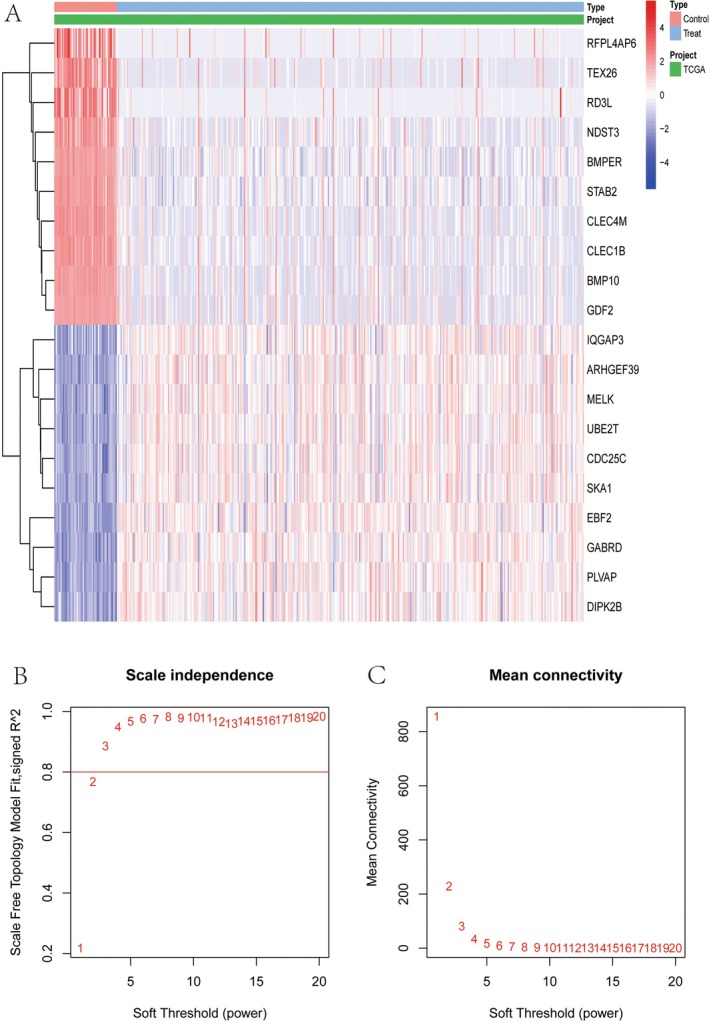
Gene expression and network analysis. (A) Gene expression heatmap: Displays the expression levels of various genes in “Control” and “Treat” group samples, with colours ranging from blue to red indicating low to high expression. (B) Scale independence plot: The relationship between soft‐thresholding power and the scale‐free topology fit index used to select appropriate network parameters. (C) Mean connectivity plot: Illustrates the relationship between soft‐thresholding power and mean connectivity, ensuring network connectivity.

Gene expression levels across various samples are displayed (HCC tissues GSE25097, DR samples GSE160306), with a colour gradient from blue (low expression) to red (high expression) representing the variation in gene expression. The samples are grouped into ‘Control’ and ‘Treat’ categories, as well as a project classification labelled “TCGA” (Figure 5A). Dendrograms on the side cluster genes based on similarities in expression patterns, suggesting potential functional relationships or co‐regulation (Figure 5A). The scale‐free topology fit index is plotted as a function of the soft‐thresholding power, with the *y*‐axis representing the fit index and the *x*‐axis representing the power. The optimal soft‐thresholding power is typically selected when the fit index approaches or exceeds 0.8, indicating a scale‐free network (Figure 5B). Additionally, the mean connectivity is plotted against the soft‐thresholding power, helping to ensure that the network remains connected while maintaining scale‐free topology characteristics (Figure 5C).

### 3.6 Weighted Gene Co‐Expression Network Analysis (WGCNA)

**FIGURE 6:**
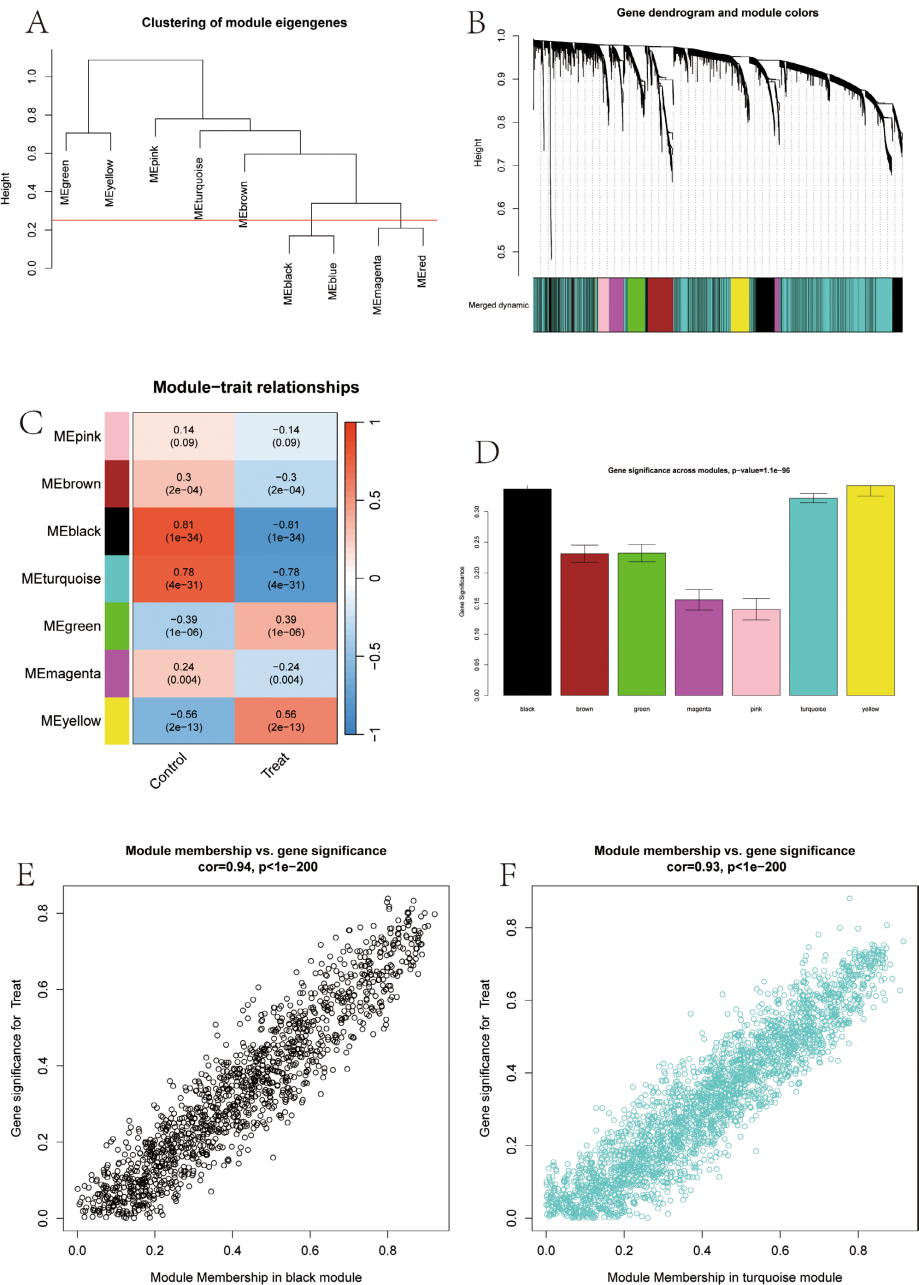
Weighted gene co‐expression network analysis (WGCNA). (A) Module feature gene clustering: Cluster analysis shows modules with similar expression patterns. (B) Gene tree diagram and module colour: Genes are divided into co‐expressed modules of different colours. (C) Module trait relationship: Heat map displays the correlation between modules and external traits (such as Control and Treat). (D) Gene significance in modules: A bar chart displays the average significance of genes and traits in each module. (E) Black module: Scatter plot displays the correlation between module membership relationships and gene significance within the black module. (F) Blue‐green module: Similar to E, but specific to the blue‐green module.

Dendrogram clustering of modules based on their eigengenes, which represent the first principal component of each module, reveals groups of modules with similar expression patterns in DR samples (GSE160306) (Figure 6A). A hierarchical clustering dendrogram of genes is presented, with branches cut to form distinct modules, each assigned a unique colour to indicate groups of co‐expressed genes (Figure 6B). The correlation between module eigengenes and external traits, such as Control versus Treat, is displayed, with colour intensity and values reflecting the strength and direction of these correlations, alongside significant *p* (Figure 6C). The average gene significance for each module is shown, highlighting the modules most strongly associated with the trait of interest (Figure 6D). The relationship between module membership (how well genes belong to the black module) and gene significance for the trait is illustrated, with a high correlation indicating that genes highly connected in the module are also highly significant for the trait (Figure 6E). Similarly, for the turquoise module, the correlation between module membership and gene significance for the trait is displayed (Figure 6F).

### 3.7 Machine Learning Models and Differential Gene Expression

**FIGURE 7:**
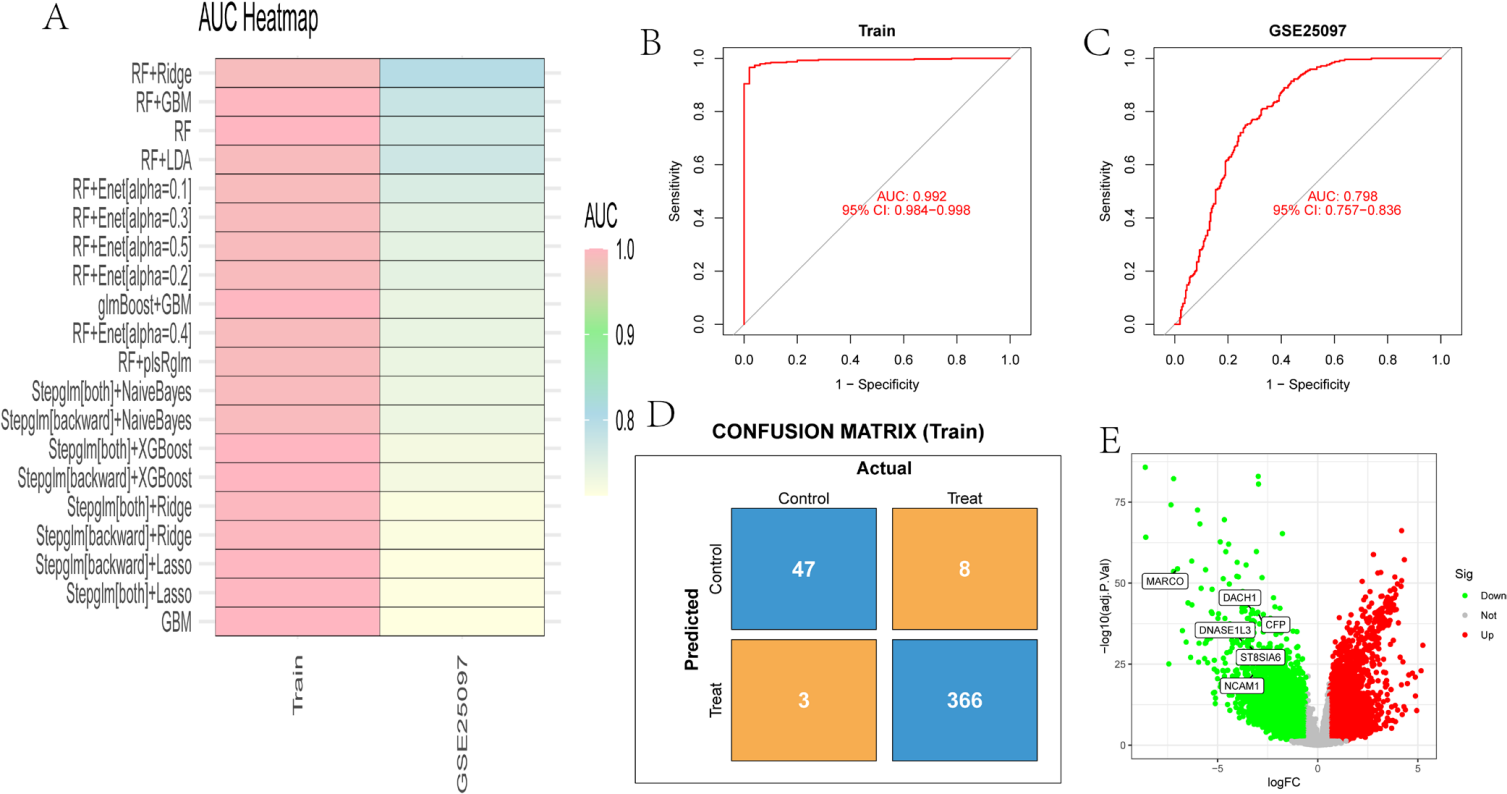
Machine learning models and differential gene expression. (A) AUC heatmap: Displays AUC values for different models on the training and GSE25097 datasets, assessing model performance. (B) ROC curve for training Data: AUC is 0.992, indicating excellent performance on the training set. (C) ROC curve for GSE25097 dataset: AUC is 0.798, showing good performance but lower than the training set. (D) Confusion matrix (Train): Shows classification accuracy of the model on the training set. (E) Volcano plot: Illustrates differential gene expression, highlighting significantly up‐regulated and downregulated genes.

The Area Under the Curve (AUC) values for various machine learning models across two datasets, GSE281110 (HCC tissue, Training) and GSE25097 (HCC tissue, Validation), are shown, with models including combinations such as RF (Random Forest) with Ridge, GBM and others. Solid research methodology in this field was referenced [22, 23, 24]. The AUC values reflect model performance, with higher values indicating better predictive accuracy, approaching 1 (Figure 7A). The receiver operating characteristic (ROC) curve illustrates model performance on the training dataset, with an AUC of 0.992 and a 95% confidence interval of 0.984 to 0.998, demonstrating excellent predictive ability (Figure 7B). The AUC for the ‘GSE25097’ dataset is 0.798, with a 95% confidence interval of 0.757–0.836, indicating good but less robust performance compared to the training data (Figure 7C). The confusion matrix displays true positives, false positives, and false negatives, providing insight into the model's classification accuracy (Figure 7D). Differential gene expression is visualised, with log fold change (logFC) on the x‐axis and negative log *p*‐value on the y‐axis. Red dots represent significantly upregulated genes, green dots represent downregulated genes, and grey dots indicate non‐significant genes. Labelled genes with notable changes are highlighted, suggesting their potential importance in the study context (Figure 7E).

### 3.8 Comprehensive Analysis Involving Gene Expression, Cell Type Distribution

**FIGURE 8:**
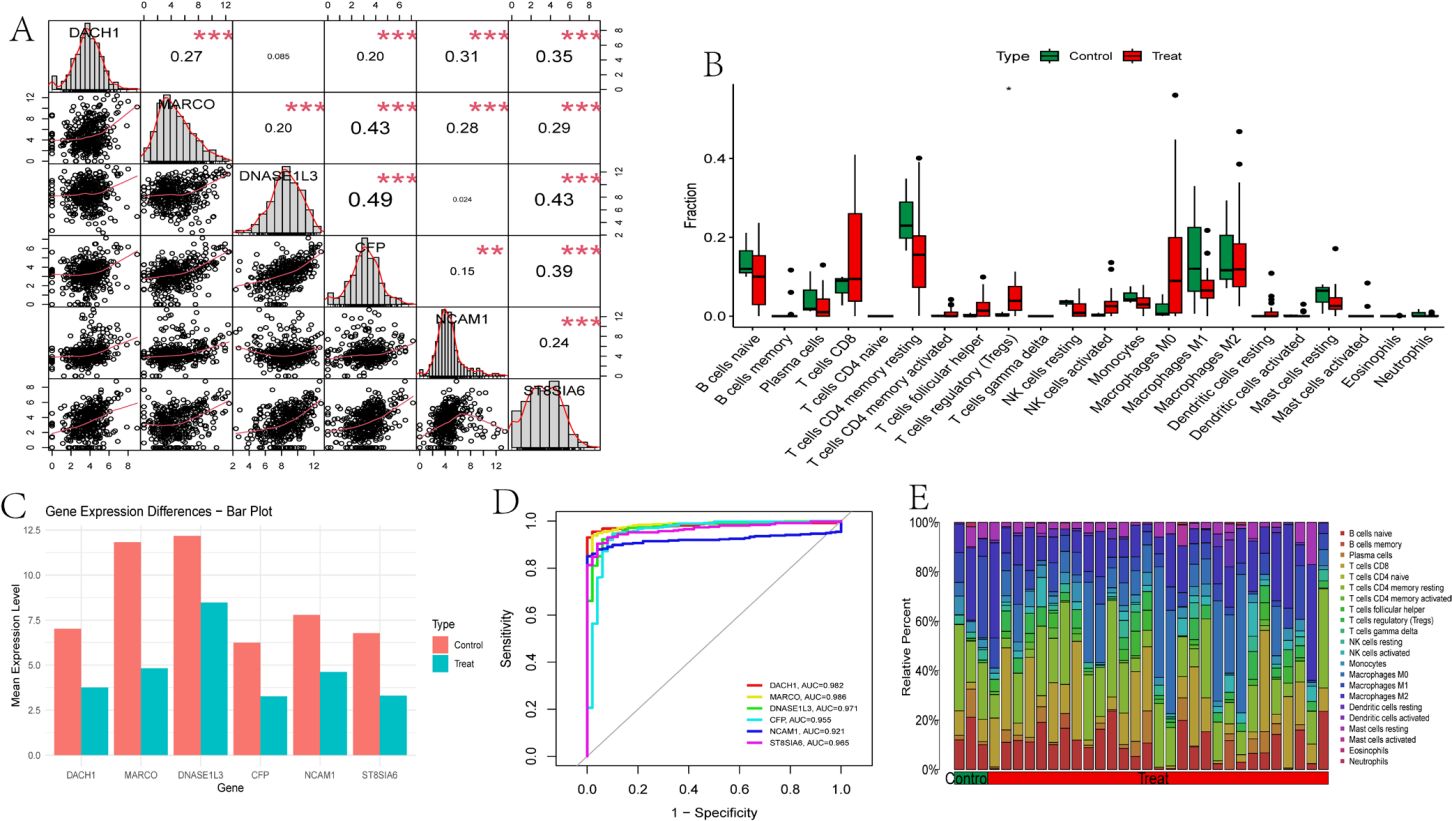
Comprehensive analysis involving gene expression and cell type distribution. (A) Correlation matrix: Displays the correlation and significance between genes. (B) Cell type distribution: Compare the proportion of different immune cell types in the “Control” and “Treat” groups. (C) Gene expression differences: A bar chart displays the average expression differences of specific genes between two groups. (D) ROC curve: The higher the AUC value, the better the predictive performance of different genes or models. (E) Cell type stacking diagram: The relative proportion changes of different cell types in each sample.

Pairwise correlations between genes, such as DACH1, MARCO and DNASE1L3, are displayed in scatter plots with histograms along the diagonal. Correlation coefficients are provided, with statistical significance indicated by asterisks (Figure 8A). A comparison of immune cell type fractions between the ‘Control’ and ‘Treat’ groups reveals significant differences, highlighting potential changes in immune response (Figure 8B). Mean expression levels of specific genes are compared between the ‘Control’ and ‘Treat’ groups, with observed differences suggesting genes that may play important roles in treatment response (Figure 8C). ROC curves for different genes and models assess their predictive performance, with AUC values indicating how effectively each gene or model differentiates between groups. Higher AUC values suggest better predictive accuracy (Figure 8D). The relative proportions of various cell types in each sample are illustrated, with differences between ‘Control’ and ‘Treat’ groups providing insights into changes in the immune landscape (Figure 8E).

### 3.9 Analysis of Immune Cell Correlations and Their Associations With Specific Traits or Conditions

**FIGURE 9:**
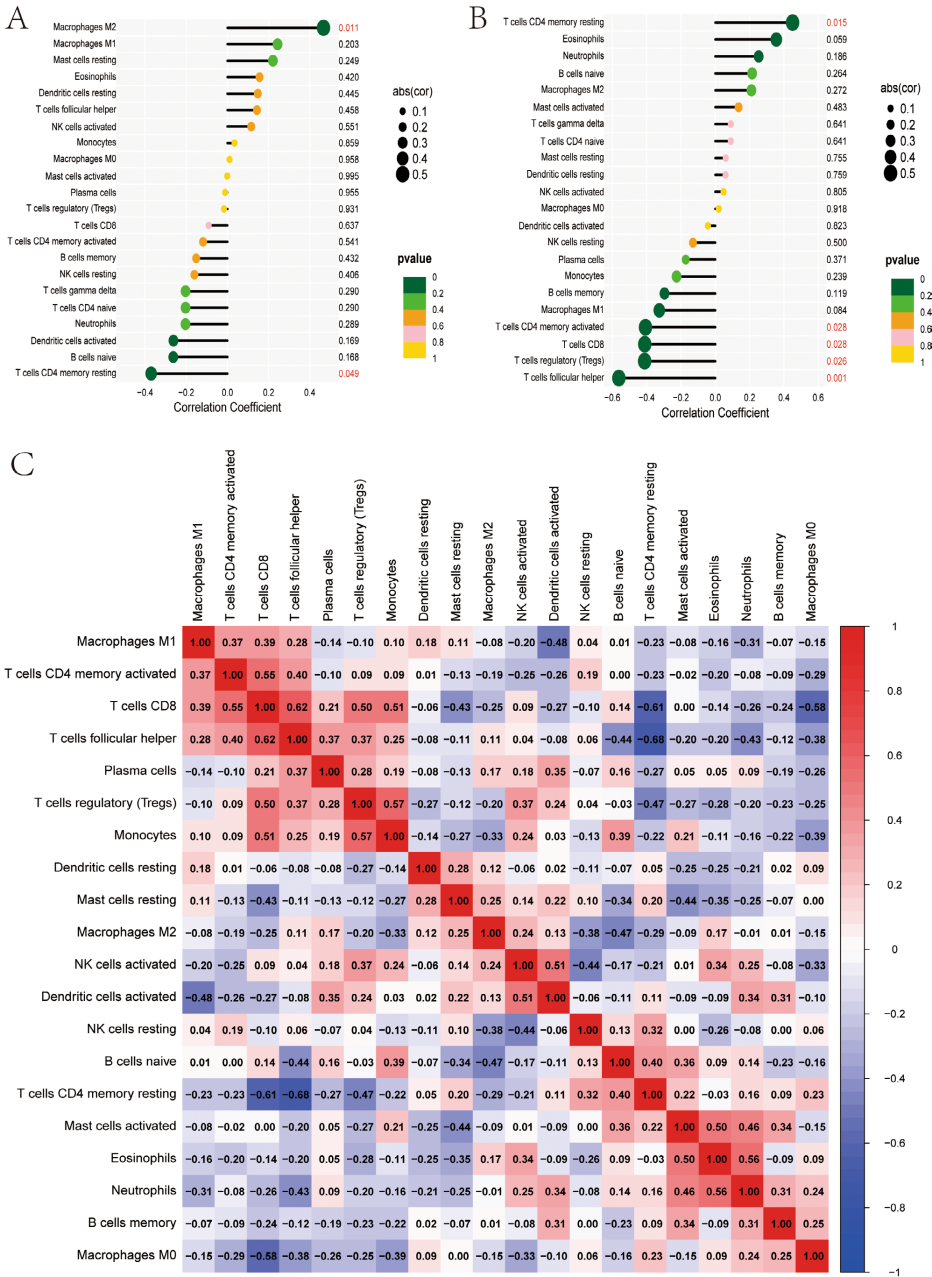
Analysis of immune cell correlations and their associations with specific traits or conditions. (A) Feature 1: Display the correlation between immune cells and Feature 1. (B) Feature 2: Display the correlation between immune cells and Feature 2. (C) Cell correlation heatmap: Displays the positive and negative correlations between immune cells.

The correlation coefficients between various immune cell types and a specific trait are displayed, with the size of the circles representing the strength of the correlation. The colour of the circles indicates the statistical significance, with darker colours corresponding to lower *p* (Figure 9A). A similar analysis is performed for a different trait, where correlation coefficients, circle sizes and colours convey the same information regarding the relationship and significance (Figure 9B). Pairwise correlations between different immune cell types are presented, with red indicating positive correlations and blue representing negative correlations. The colour's intensity reflects the correlation's strength (Figure 9C).

### 3.10 The Correlation Between DACH1 Gene Expression and Various Immune Cell Types

**FIGURE 10:**
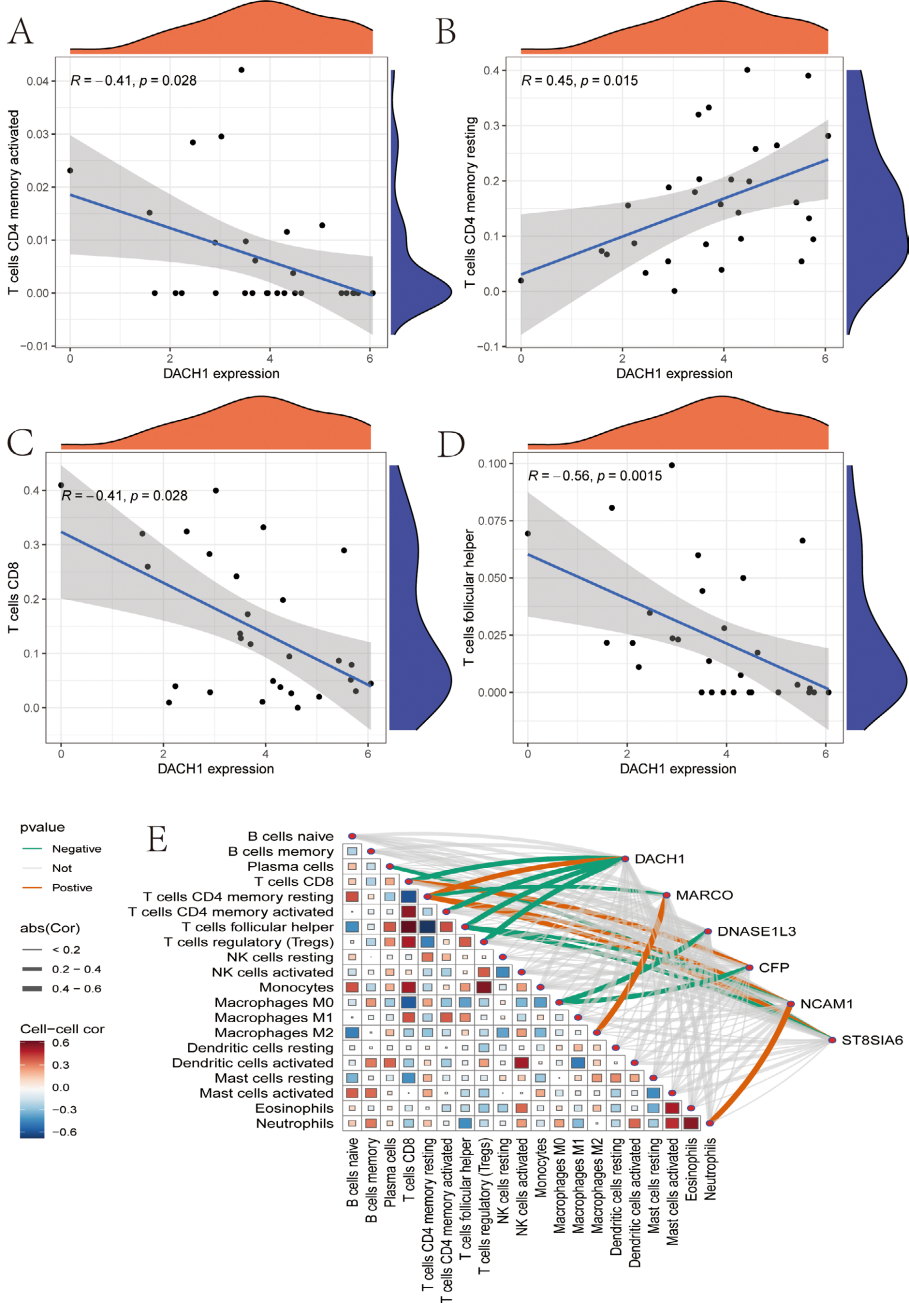
The correlation between DACH1 gene expression and various immune cell types. (A–D) Correlation diagram: The positive and negative correlations between DACH1 and different immune cells and the negative correlation with CD8 T cells. (E) Gene cell network: Displays significant interactions between genes and immune cells, with lines indicating correlation strength and direction.

The relationship between DACH1 expression and specific immune cells is illustrated. A negative correlation with activated CD4 memory T cells is observed (*R* = −0.41, *p* = 0.028) (Figure 10A). A positive correlation is found with resting CD4 memory T cells (*R* = 0.45, *p* = 0.015) (Figure 10B). DACH1 expression also shows a negative correlation with CD8 T cells (*R* = −0.41, *p* = 0.028) (Figure 10C) and a stronger negative correlation with follicular helper T cells (*R* = −0.56, *p* = 0.0015) (Figure 10D). A heatmap further displays correlations between different immune cells, with lines connecting genes (e.g., DACH1, MARCO) to immune cells, indicating significant interactions. The thickness and colour of the lines represent the strength and direction of these correlations, with red indicating positive and blue indicating negative correlations (Figure 10E).

### 3.11 Single‐Cell Dimension Reduction Cluster Analysis of DR

**FIGURE 11:**
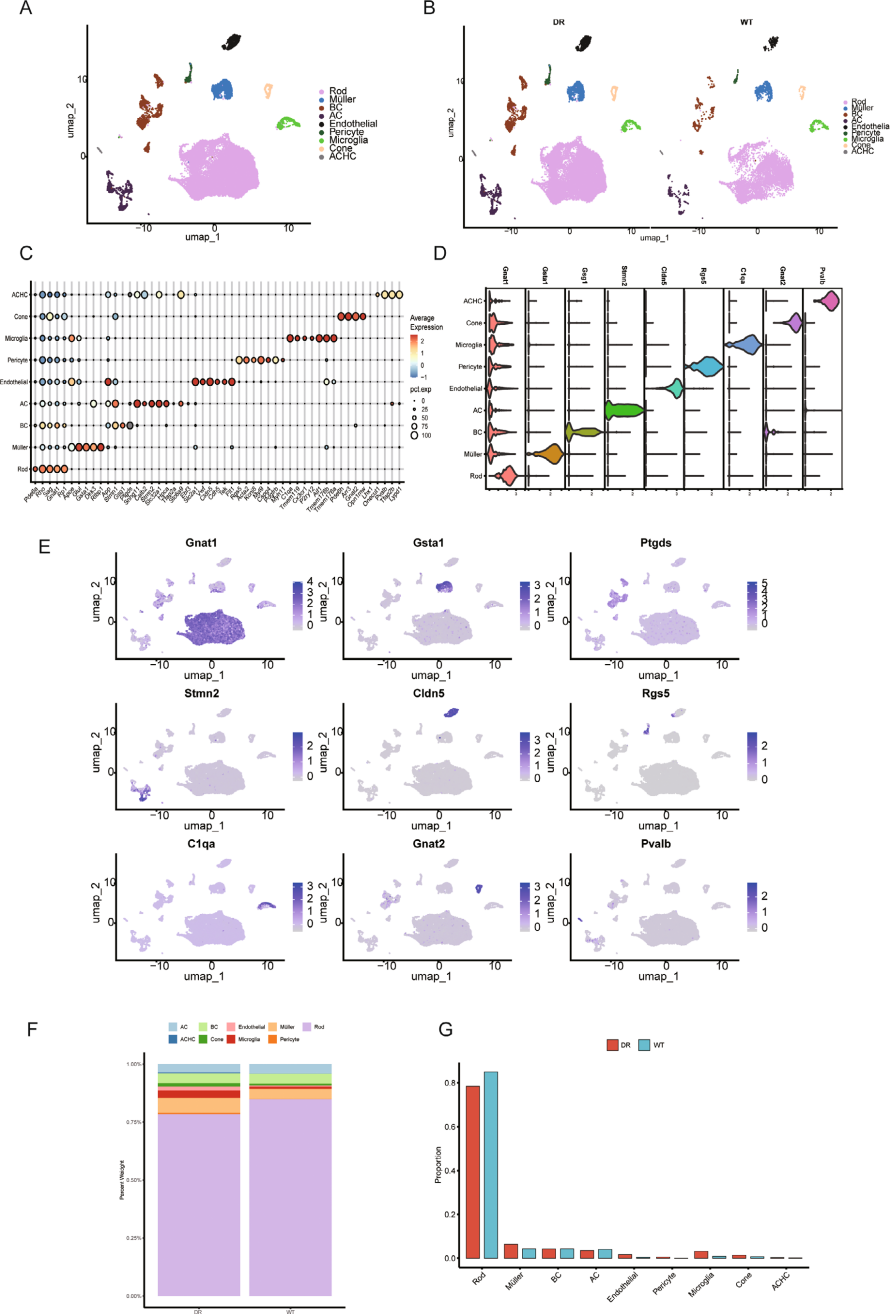
Single‐cell dimension reduction cluster analysis of DR. (A) Cell annotation UMAP display; (B) Cell annotated UMAP disease and control group display; (C) Bubble map of marker gene. (D) Violin map of the cell marker gene. (E) Scatter map of marker genes in cells, where one figure represents one gene. (F) Cell proportion bar stack diagram. (G) Cell proportion bar diagram.

Single‐cell data from GSE209872 (DR samples) were processed through dimensionality reduction and clustering, followed by manual annotation, resulting in the identification of nine distinct cell types: Rod, Müller, BC (Bipolar Cells), AC (Amacrine Cells), Endothelial, Pericyte, Microglia, Cone and ACHC (Figure 11A). The distribution of cells from the disease and control groups is shown, with separate visualisations for each group (Figure 11B). Marker gene expression, used for cell annotation, is represented in bubble plots, violin plots and scatter plots (Figure 11C–E). Analysis of cell proportions revealed significant changes between groups. Specifically, Rod and AC cells showed a marked decrease in the disease group, while Müller, Endothelial, Microglia and Cone cells exhibited increased proportions in the disease group (Figure 11F,G).

### 3.12 Impact of Ferritinophagy Gene on Single Cells of DR

**FIGURE 12:**
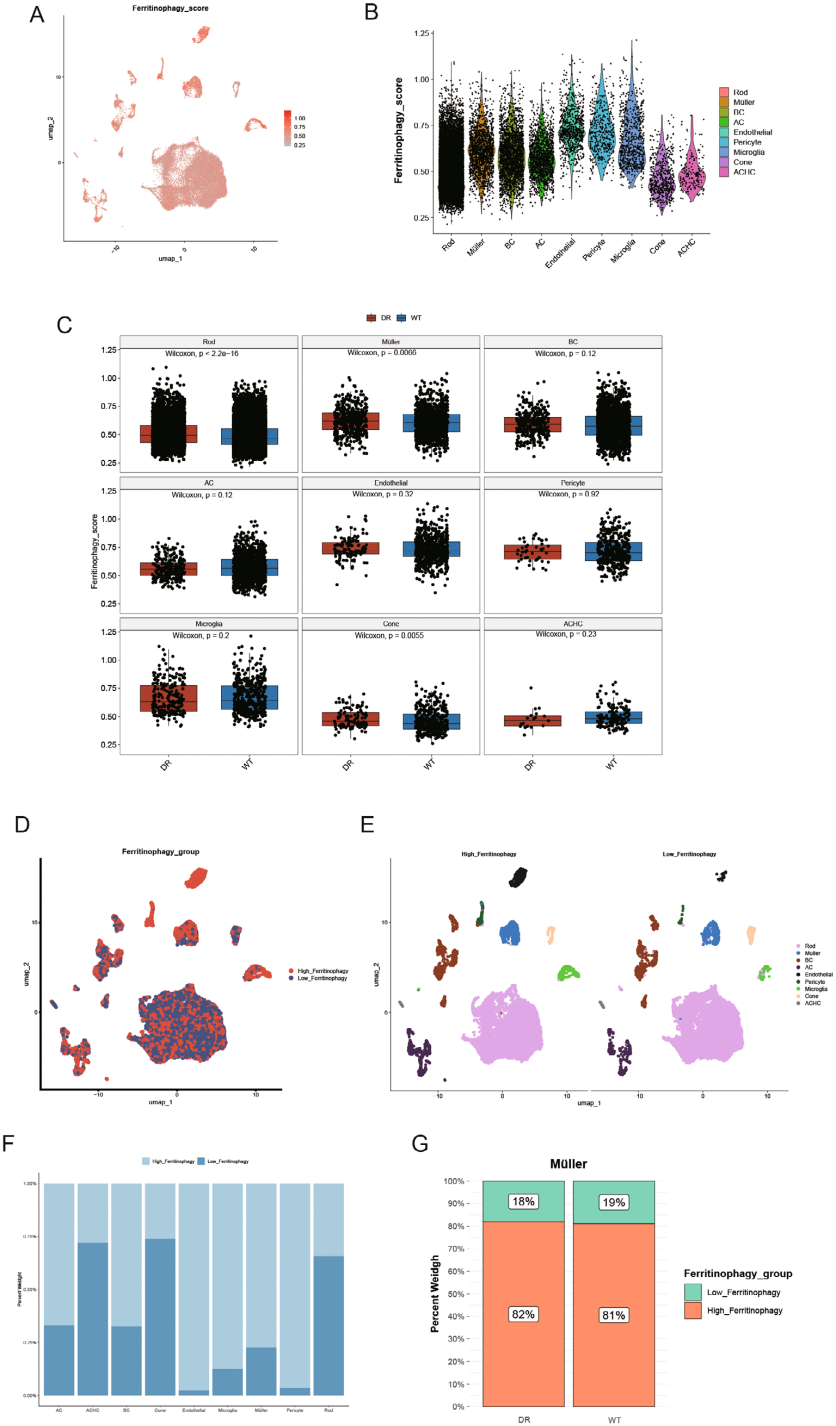
Impact of Ferritinophagy gene on single cells of DR. (A) UMAP display of Ferritinophagy score in cells; (B) Ferritinophagy scores were shown in different cells. (C) Box diagram for analysis of differences in Ferritinophagy scores among different cell disease controls, with one figure representing one cell; (D, E) UMAP shows the grouping of high Ferritinophagy and low Ferritinophagy in cells. (F) Histogram of the proportion of different cells in the high‐low Ferritinophagy group; (G) The proportion of the high‐low Ferritinophagy group in Müller cells in the disease control group was histogram stacked.

To investigate the expression of Ferritinophagy genes in major cell types across disease and control groups, the ssGSEA method was used to calculate the Ferritinophagy_score for each cell. The results were visualised using UMAP (Figure 12A). Cone and ACHC cells exhibited lower Ferritinophagy scores than other cell types (Figure 12B). Differential analysis of Ferritinophagy scores between the disease and control groups (Figure 12C) revealed that Müller, Rod and Cone cells showed significant differences, with higher Ferritinophagy scores observed in the disease group compared to the control group (*p* < 0.05). Based on the median Ferritinophagy score (0.499), cells were categorised into high and low Ferritinophagy groups. UMAP visualisation of these groups is shown in the results (Figure 12D). The proportion of high and low Ferritinophagy cells across different types was analysed, with Endothelial, Microglia, Müller and Pericyte cells predominantly represented in the high Ferritinophagy group, while ACHC, Cone and Rod cells were more abundant in the low Ferritinophagy group (Figure 12D–F). Furthermore, comparisons of Ferritinophagy scores between the disease and control groups within the high and low Ferritinophagy categories (Figure 12G) indicated that Müller cells in the DR group had a significantly greater proportion of high Ferritinophagy scores than the low‐score group. In contrast, Rod and Cone cells exhibited the opposite trend (Rod and Cone cell results are shown in Figure 12A).

### 3.13 Membrane Protein‐Mediated Ferritinophagy Communication

**FIGURE 13:**
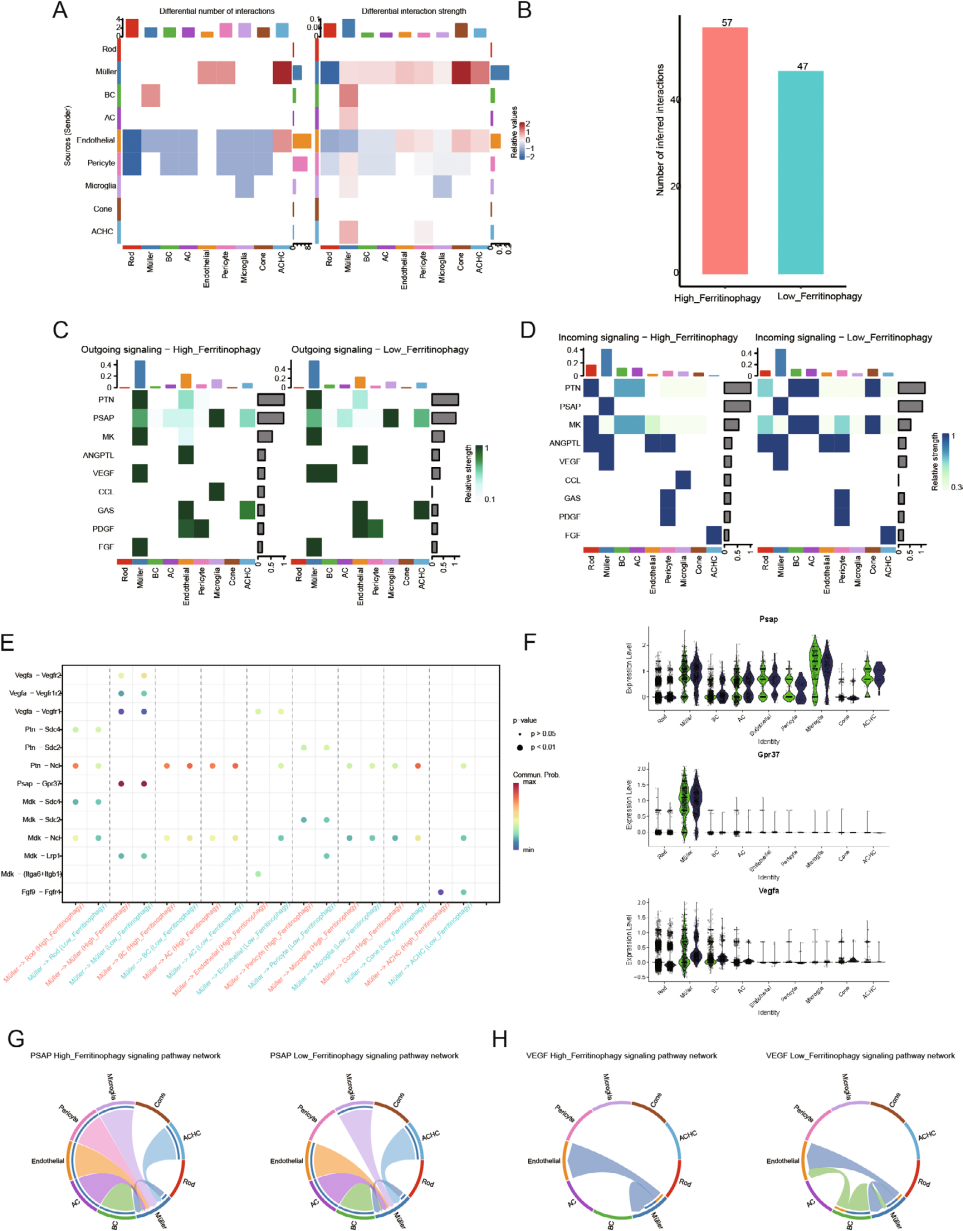
Membrane protein‐mediated Ferritinophagy communication. (A) Compared with the heat map of the number of cell ligand‐receptor pairs in the high‐Ferritinophagy group and the low‐Ferritinophagy group, the closer the colour is to red, the higher the frequency and intensity of cell communication in the high‐Ferritinophagy group, and the closer the colour is to blue, the higher the frequency and intensity of cell communication in the low‐Ferritinophagy group. (B) Bar chart of cell communication frequency in the disease group and control group; (C, D) heat map of the pathway of cell generation communication information (efferent and afferent) in high‐rated and low‐rated groups. The abscess represents the cell, and the ordinate represents the name of the communication pathway. (E) heat maps of up‐regulated and down‐regulated receptor pairs of cells in high and low‐rated groups; (F) expression of ligand‐receptor genes belonging to membrane proteins; (G) the PSAP signalling pathway was shown in the chord diagram between different cells; (H) the Vegf signalling pathway between different cells was shown in the chord diagram. Different colours indicated the communication between different cells, and the width indicated the intensity of communication.

Intercellular communication relies on complex interactions between ligands and their receptors and the subsequent activation of specific cell signalling pathways. Studying these ligand‐receptor interactions is crucial for understanding cell behaviour and complex biological processes. CellChat software was used to investigate this to predict cell communication within the high and low Ferritinophagy groups, with a significance threshold of *p* < 0.05. As shown in Figure 13A, the frequency of cell communication between Müller cells and BC, AC, Endothelial, Pericyte, Microglia, Cone and ACHC cells was greater in the high Ferritinophagy group compared to the low group. Conversely, communication between Endothelial and Rod, Müller, BC, AC and Müller–Rod cells was less frequent in the high group than in the low group. Additionally, the overall frequency of intercellular communication in the high Ferritinophagy group was higher than in the low group, as depicted in Figure 13B.

To further elucidate the complex network of intercellular interactions and their implications, the communication network was systematically analysed to identify the dominant signalling roles of each cell type—both as transmitters and receivers. This analysis highlights the signalling contributions of each cell type to outgoing and incoming signal transmission in different groups. The results of efferent and afferent signalling pathways are presented in Figure 13C,D. Notably, signalling pathways such as Psap and MK were enhanced in the high Ferritinophagy group compared to the low group, while VEGF signalling was more pronounced in the low Ferritinophagy group, suggesting these pathways could play a significant role in disease progression.

The major differences in ligand‐receptor pairs between cells were concentrated in Psap−Gpr37, Vegfa−Vegfr1, Vegfa−Vegfr2, PTN − NCl and MDK − NCl, among others. Differential analysis of ligand‐receptor pairs revealed that Psap, Gpr37 and Vegfa are membrane‐bound proteins. Psap gene expression was observed across most cell types, with Gpr37 predominantly expressed in Müller cells and Vegfa highly expressed in Rod, Müller, and BC cells. The Psap and VEGF signalling pathways were further visualised. Figure 13G shows the Psap signalling pathway, primarily present in Müller cells and other cell types, and is involved in cellular processes such as proliferation, differentiation, apoptosis and intercellular communication. The VEGF signalling pathway, shown in Figure 13F, is a vascular endothelial growth factor pathway associated with blood vessel progression in DR. In the high Ferritinophagy group, communication through this pathway was mainly observed in Müller and BC cells, whereas in the low Ferritinophagy group, it was more prominent in Müller, Endothelial and BC cells. Further analysis suggests that Müller cells may be pivotal in mediating the effects of membrane‐bound proteins in the progression of the disease.

### 3.14 Analysis of Subsets of Ferritinophagy Related Cells

**FIGURE 14:**
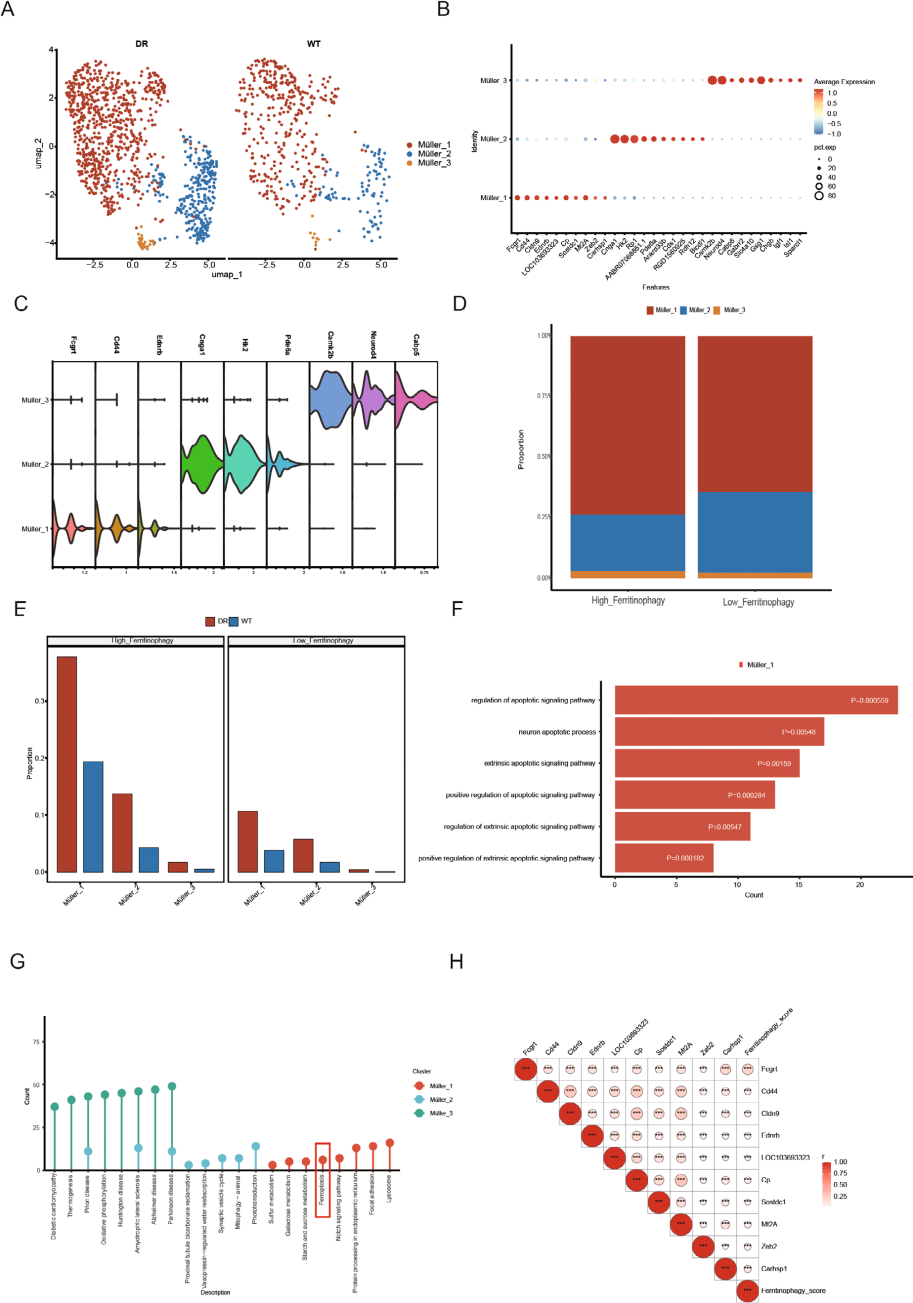
Analysis of Subsets of Ferritinophagy related cells. (A)UMAP display of Müller subpopulation heterogeneity; (B) marker gene bubble map of the Müller subgroup; (C) marker gene violin diagram of Müller subgroup; (D) Müller subpopulation in high and low ranking group proportion histogram; (E) Histogram of the proportion of Müller subgroup in the disease control group; (F) Bar diagram of autophagy‐related pathways for GO enrichment analysis of subpopulation characteristic genes; (G) Rod diagram for KEGG enrichment analysis; (H) Heat map of correlation between top 10 characteristic genes of Müller1 subgroup and Ferritinophagy score.

To investigate the heterogeneity of Müller cells, the cells identified in the previous step were subjected to dimensionality reduction and cluster analysis. Based on the optimal resolution, a resolution of 0.2 was selected, resulting in the identification of three distinct Müller cell subgroups: Müller1, Müller2 and Müller3 (Figure 14A). To identify genes that differentiate these Müller cell subtypes, the *FindAllMarkers* function was applied with parameters set to min.pct = 0.2 only. Pos = TRUE, and logfc.threshold = 0.5. The results revealed that Müller1 cells highly expressed genes such as Fcgrt, Cd44, Ednrb, Eci2, and others; Müller2 cells were characterised by high expression of Cnga1, Hk2, Pde6a, among others; and Müller3 cells expressed genes such as Camk2b, Neurod4, Cabp5 and others (Figure 14B,C). A full list of differentially expressed genes is provided in the Table S5.

Proportion analysis results (Figure 14D,E) showed that the proportion of the Müller1 subgroup in the high Ferritinophagy group was significantly higher than in the low Ferritinophagy group, while the proportion of Müller2 cells was increased in the low Ferritinophagy group. Notably, the proportion of Müller1, Müller2 and Müller3 cells was higher in the disease group compared to the control group.

Gene Ontology Biological Process (GOBP) enrichment analysis (Figure 14F) indicated that the Müller1 subgroup was significantly enriched in pathways related to the regulation of apoptosis, including positive regulation of exogenous apoptosis signalling, neuronal apoptosis and apoptosis signalling pathways.

KEGG enrichment analysis (Figure 14G) further revealed that Müller1 cells were enriched in pathways such as sulfur metabolism, galactose metabolism, starch and sucrose metabolism, Ferroptosis, Notch signalling, protein processing in the endoplasmic reticulum and adhesive plaque lysosome. The Müller2 subgroup was enriched in pathways related to mitogenic signalling, Parkinson's disease, amyotrophic lateral sclerosis and phototransduction. Müller3 cells were enriched in pathways associated with diabetic cardiomyopathy, heat generation, prion disease and oxidative phosphorylation.

Finally, the top 10 feature genes with the greatest expression differences in the Müller1 subgroup were selected, and their correlation with the Ferritinophagy score was calculated. The results showed a significant positive correlation between these feature genes and the Ferritinophagy score (*p* < 0.05).

### 3.15 Potential Target Membrane Protein‐Mediated Iron‐Autotropic Key Subtype Gene Drugs

**FIGURE 15:**
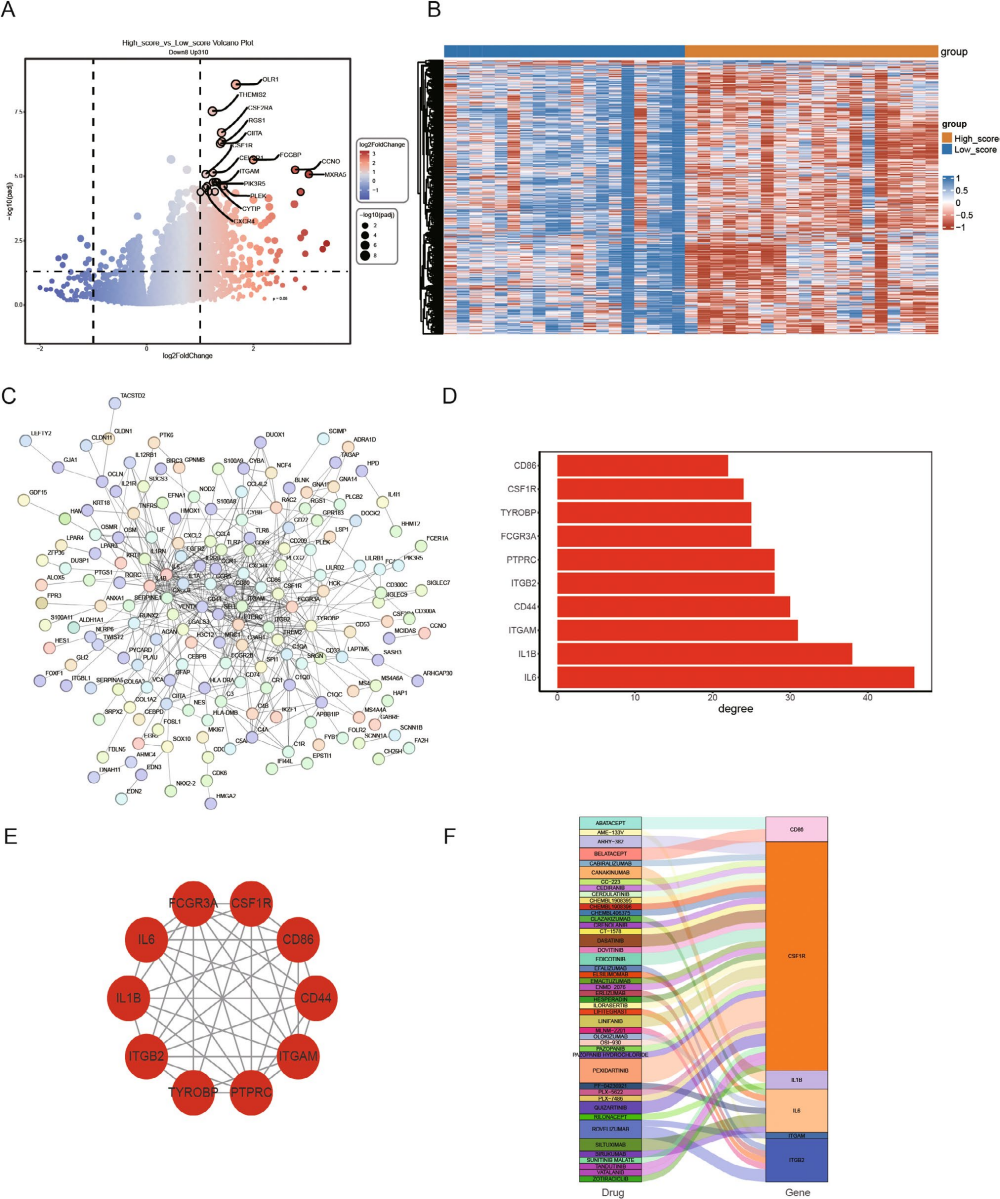
Potential target membrane protein‐mediated iron‐autotropic key subtype gene drugs. (A) Volcano map of differentially expressed genes between high‐rated subgroup and low‐rated subgroup of Bulk cohort; (B) heat map of differential genes; (C) differential gene protein interaction Network (PPI); (D) protein interaction network top10hub gene connectivity display; (E) hub gene network diagram; (F) target drug prediction of hub gene.

To explore the differential expression of genes based on Müller1 subgroup characteristics, ssGSEA was applied to the Bulk cohort to score and calculate samples. The samples were then categorised into high and low‐score groups according to the median score. Differential expression analysis between the high and low‐score groups was conducted using DESeq2 software. Three hundred eighteen differentially expressed genes were identified, with 310 genes upregulated in the high‐score group and eight downregulated (Figure 15A). A volcano plot generated using *ggplot2* (version 3.5.0) illustrates the differential expression of these genes. Heatmaps created with *ComplexHeatmap* (version 2.18.0) were used to visualise the expression patterns of upregulated and downregulated genes (Figure 15B).

An interaction network of the 318 differentially expressed gene proteins was constructed using the STRING database, revealing a network of 173 nodes and 517 edges (Figure 15C). Hub genes, defined as genes with critical roles in the network, were identified based on the degree index of each node. The top 10 genes with the highest degree were selected as hub genes, with the results showing that IL‐6 had the highest degree, followed by IL‐1B, suggesting that these hub genes play a more significant role in the network than other genes (Figure 15D). Further investigation revealed that these 10 hub genes are primarily involved in inflammation and immunity.

To examine potential therapeutic targets, the protein–protein interaction network of the 10 hub genes was constructed (Figure 15E). Additionally, we utilised the DGIDB database (https://dgidb.org/) to predict potential target drugs for these hub genes. A relationship map between genes and drugs was plotted using *ggplot2*, showing that 45 target drugs were linked to the 10 hub genes, with six drugs associated with IL‐6. Notably, drugs such as SILTUXIMAB, CLAZAKIZUMAB, SIRUKUMAB, ELSILIMOMAB, PF‐04236921, and OLOKIZUMAB were found to target the CSF1R gene and other hub genes (Figure 15F).

### 3.16 Validation of Central Hub Genes NCOA4, GPX4, SLC7A11 and FRHGs

**FIGURE 16:**
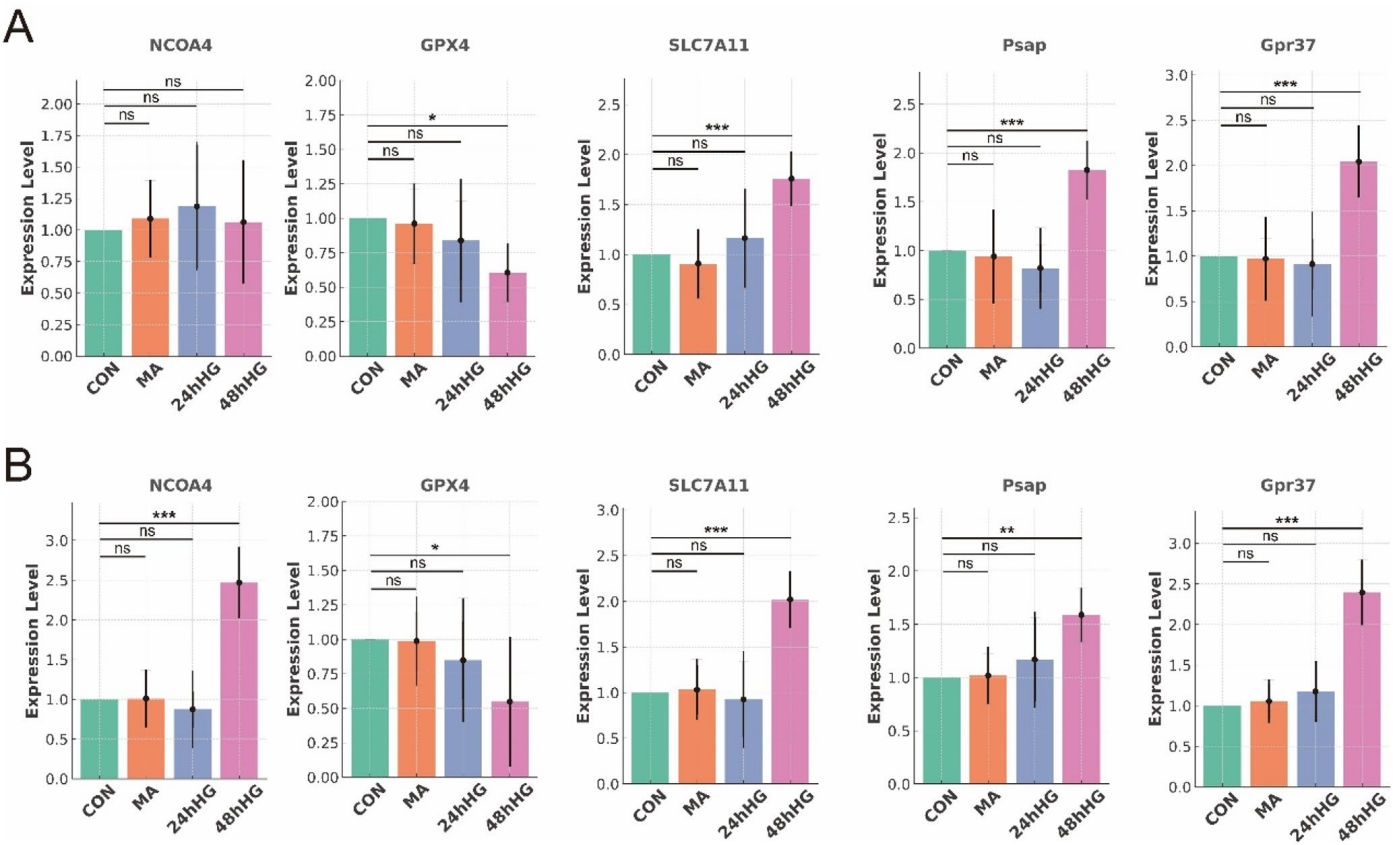
Validation ferritin phagocytosis‐related genes NCOA4, GPX4, SLC7A11 PSAP and GPR37 in Müller and HCC cells. (A) External validation of NCOA4, GPX4, SLC7A11 PSAP, and GPR37 mRNA levels by qRT‐PCR in untreated (control group), mannitol hypertonic group, 24 h high glucose treated group and 48 h high glucose treated group in Müller cells (*n* = 5). (B) External validation of NCOA4, GPX4, SLC7A11 PSAP, and GPR37 mRNA levels by qRT‐PCR in untreated (control group), mannitol hypertonic group, 24 h high glucose treated group and 48 h high glucose treated group in HepG2 (HCC) cells (*n* = 5). *p < 0.05; **p < 0.01; ***p < 0.001.

To investigate the expression of key genes involved in ferritinophagy and oxidative stress response, we cultured RMC‐1 cells under normal glucose conditions (CON), mannitol‐treated conditions (MA), and high glucose conditions for 24 h (24hHG) and 48 h (48hHG). The expression levels of genes NCOA4, GPX4, SLC7A11, Psap, Gpr37 and VegfA were assessed using qRT‐PCR. Our results indicated significant changes in the expression of ferritinophagy and oxidative stress markers in response to high glucose treatment. In the 48hHG group, a significant upregulation of NCOA4 was observed (2.8‐fold increase compared to CON, *p* = 0.02), while both GPX4 and SLC7A11 expression levels were significantly downregulated (1.5‐fold decrease, *p* = 0.03 and 2.1‐fold decrease, *p* = 0.01, respectively). These findings highlight the dysregulation of the antioxidant response in the high glucose environment. Further, the expression levels of Psap, Gpr37 and VegfA were significantly increased in the 48hHG group compared to the CON group (Psap: 3.2‐fold increase, *p* = 0.005; Gpr37: 2.7‐fold increase, *p* = 0.01; VegfA: 2.5‐fold increase, *p* = 0.02). These changes suggest a potential role of these genes in the inflammatory and vascular alterations observed in diabetic conditions. In addition, the qRT‐PCR analysis confirmed that the expression of key inflammatory markers, including IL‐6, IL‐1β, ITGAM and ITGβ2, was significantly upregulated in RMC‐1 cells after 48 h of high glucose exposure (IL‐6: 2.1‐fold increase, *p* = 0.04; IL‐1β: 2.4‐fold increase, *p* = 0.03; ITGAM: 2.3‐fold increase, *p* = 0.03; ITGβ2: 2.2‐fold increase, *p* = 0.03). These results were consistent with our bioinformatics analysis (Figure 16), further supporting the role of high glucose in inducing a pro‐inflammatory state (Figure 16).

## 4 Discussion

Diabetes is not only an independent risk factor for liver cancer but may also share common molecular mechanisms with DR. Inflammatory responses and VEGF are key factors in both conditions. Chronic inflammation promotes liver cancer progression and is closely linked to the pathogenesis of DR, where it exacerbates retinal vascular dysfunction. VEGF, a major mediator of angiogenesis, plays a critical role in both liver cancer and DR, contributing to tumour growth in HCC and pathological neovascularization in DR [25, 26]. Ferritinophagy, a selective autophagy process, also regulates iron metabolism and oxidative stress in both diseases. Ferritinophagy helps maintain iron homeostasis and ROS production in liver cancer, supporting tumour progression. In DR, dysregulated Ferritinophagy leads to iron accumulation in the retina, contributing to oxidative stress and possibly promoting ferroptosis. These shared mechanisms suggest that targeting Ferritinophagy could offer therapeutic benefits for both DR and HCC, particularly in diabetic patients at risk for both conditions.

Diabetic retinopathy is a severe complication of diabetes, primarily resulting from poor blood sugar control, leading to retinal vascular and neurological damage, and ultimately causing visual impairment or blindness through complex pathophysiological mechanisms. Recent studies emphasise iron homeostasis regulation's crucial role in disease progression and cellular function [27, 28]. Ferritinophagy, a process that degrades ferritin and releases iron, acts as an essential feedback mechanism for intracellular iron regulation. Its activation increases the available iron content, influencing sensitivity to ferroptosis—a form of cell death linked to oxidative stress. This mechanism bridges metabolism, disease progression, immune responses, and potential therapeutic targets, suggesting that Ferritinophagy is critical in DR and liver cancer, where iron dysregulation plays a pivotal role in tumour progression [29, 30].

Ferritinophagy is emerging as a promising research direction in ophthalmology, with potential for targeted therapies. Studies indicate that α‐synuclein disrupts ferritin autophagy, increasing ferritin expression in retinal and retinal pigment epithelium (RPE) cells, contributing to retinal degeneration in conditions like Parkinson's. Additionally, research has shown that ferroptosis plays a role in ischemic reperfusion‐induced retinal injury, with Ferritinophagy mediated by 70 kDa androgen receptor‐related proteins as a key mechanism in ferroptosis in retinal ganglion cells. These findings suggest that targeting Ferritinophagy could offer therapeutic benefits in retinal diseases like DR and liver cancer, where iron dysregulation and ferroptosis are critical in tumour progression.

In this study, we first performed dimensionality reduction and cluster analysis on single‐cell data to identify cellular changes in DR and HCC. Our analysis revealed that Rod and AC cells decreased in the disease group, while Müller, Endothelial, Microglia and Cone cells showed increased proportions. This suggests a shift in the cellular composition between DR and HCC. Importantly, based on the expression of Ferritinophagy‐related genes, we found a significantly higher Ferritin deposition score in Müller cells in the DR group compared to the control group, which aligns with the findings in liver cancer. These results highlight the role of Müller cells in both diseases, particularly in their involvement in Ferritinophagy, a process linked to iron homeostasis and oxidative stress.

Ferritinophagy, a selective autophagy process that regulates iron metabolism, plays a pivotal role in both DR and HCC. In DR, dysregulated Ferritinophagy leads to iron accumulation in Müller cells, contributing to oxidative stress and potentially promoting ferroptosis. In liver cancer, Ferritinophagy is critical for regulating cellular iron levels and reactive oxygen species (ROS), with its dysregulation fueling tumour progression. This overlap suggests that both diseases share a common pathological mechanism related to iron metabolism, potentially driving the progression of retinal damage in DR and the malignancy in HCC [31, 32].

Additionally, cell communication analysis revealed that central hub genes related to Ferritinophagy, such as PSAP and MK, were significantly upregulated in high‐ferritinophagy Müller cells in DR. These findings are consistent with similar upregulation of Ferritinophagy‐related genes in HCC, suggesting that these genes may serve as biomarkers for both conditions. Notably, VEGF expression, a key regulator of angiogenesis, was enhanced in the low‐Ferritinophagy Müller group, highlighting the role of Ferritinophagy in modulating the angiogenic response in both diseases.

The shared molecular pathways between DR and HCC—particularly those involving Ferritinophagy, iron metabolism, and oxidative stress—suggest that these diseases may benefit from similar therapeutic strategies targeting these pathways. Our results provide new insights into the intersection of DR and HCC and open the door for developing targeted therapies that address the underlying pathophysiological mechanisms in both diseases, with potential clinical implications for diabetic patients at risk for both conditions.

Interestingly, although we did not find a direct correlation between Müller cells and Ferritinophagy in DR at this stage, Müller cells are strongly associated with ferroptosis, a process closely linked to iron metabolism and oxidative stress. Ferritinophagy regulates intracellular iron levels and influences ferroptosis in DR and liver cancer (HCC). Theoretically, the dysregulation of Ferritinophagy could significantly contribute to the pathophysiology of DR by exacerbating retinal oxidative damage, thus implicating Ferritinophagy as a critical factor in the disease progression.

However, there remains a lack of studies on Ferritinophagy‐related membrane proteins and their involvement in inflammation and immune responses in DR. This gap presents limitations in our current understanding of the full scope of Ferritinophagy's role in DR. Our analysis is based on publicly available database studies, and further biological experiments, including clinical specimens and observations, are necessary to validate the reliability of our findings. Additionally, the sample size in this study was relatively small, and we anticipate that future research will include larger cohorts and more comprehensive single‐cell sequencing data to refine these findings further.

Moreover, our study's identification of potential therapeutic targets based on bioinformatics analysis highlights the need for in vivo and in vitro validation to confirm the efficacy of these targets in clinical settings. Future research should address the potential toxicity of these targeted therapies and evaluate whether they could cause harm to other tissues or organs. Dual validation in animal models and human trials is critical for confirming the therapeutic potential of Ferritinophagy‐related targets in both DR and HCC. Additionally, given the relatively small size of ocular organs, administering targeted therapies in eye diseases presents a unique challenge that warrants further investigation.

## 5 Conclusion

Through single‐cell sequencing and functional analysis, we identified three membrane protein genes (PSAP, GPR37 and VEGFA) involved in DR and HCC, linking them to Ferritinophagy pathways. These genes could serve as biomarkers for DR. We also identified key genes (IL‐6, IL‐1β, ITGAM, CD44 and ITGβ2) that mediate Ferritinophagy, offering potential therapeutic targets. Based on these findings, we predict drugs targeting Ferritinophagy could provide new treatments for DR. Our research highlights shared mechanisms between DR and HCC, paving the way for targeted therapies for both diseases.

## Author Contributions

**Yinan Shao:** conceptualization (equal), data curation (equal), writing – original draft (equal). **Bingfen Duan:** conceptualization (equal), data curation (equal), writing – original draft (equal). **Haotian Li:** conceptualization (equal), formal analysis (equal), validation (equal). **Xiaonan Li:** formal analysis (equal), investigation (equal), validation (equal). **Shijing Peng:** formal analysis (equal), methodology (equal), supervision (equal). **Haowen Zheng:** investigation (equal), methodology (equal), supervision (equal). **Zhipeng You:** funding acquisition (equal), project administration (equal), writing – review and editing (equal).

## Conflicts of Interest

The authors declare no conflicts of interest.

## Supporting information


Data S1.

## Data Availability

The novel findings from this research are accessible to the public. The relevant data can be accessed through the following sources: datasets GSE209872 and GSE160306, obtained from the GEO database; information from the GeneCards database; and human proteome database.
