# Supplementary material for: Target Screening and Single Cell Analysis of Diabetic Retinopathy and Hepatocarcinoma
Source: J Cell Mol Med. 2025 Apr 28;29(9):e70521. doi: 10.1111/jcmm.70521 (PMC12128161; doi:10.1111/jcmm.70521)
Supplement: Supplementary file 1 — Data S1. [file JCMM-29-e70521-s001.docx]

| **Gene_Name** | **Function** |
| --- | --- |
| Ncoa4 | Iron metabolism and ferritinophagy |
| Trim7 | Ubiquitination and protein degradation |
| Usp24 | Deubiquitination and protein stability |
| Fth1 | Iron storage and homeostasis |
| Becn1 | Autophagy regulation |
| Ftl1 | Iron storage and homeostasis |
| Ftl1l1 | Iron storage-related |
| Rnf20 | E3 ubiquitin ligase, gene expression regulation |
| Snca | Synaptic vesicle trafficking, neurodegeneration |
| Alox15 | Lipid metabolism and inflammation |
| Pcbp1 | RNA binding and iron homeostasis |
| Jun | Transcription factor, AP-1 complex |
| Mapk8 | JNK signaling, apoptosis regulation |
| Herc2 | E3 ubiquitin ligase, DNA repair |
| Elavl1 | mRNA stability and neuronal function |
| Pink1 | Mitochondrial quality control, Parkinson’s disease |
| Tnf | Pro-inflammatory cytokine |
| Nfe2l2 | Oxidative stress response, NRF2 pathway |
| Aldh1a1 | Retinoic acid biosynthesis and detoxification |
| Fbxw7 | Ubiquitin-mediated protein degradation |
| Igfbp7 | Cell growth inhibition and tumor suppression |
| Bcat2 | Branched-chain amino acid metabolism |
| Atg7 | Autophagy initiation |
| Atg16l1 | Autophagosome formation |
| Ptbp1 | RNA splicing and post-transcriptional regulation |
| Zfp36 | mRNA decay and inflammation regulation |
| Cyb561a3 | Electron transport and iron homeostasis |
| Fcgrt | Fc fragment of IgG receptor and transporter |
| Cd44 | Cell surface glycoprotein involved in cell-cell interactions, migration, and adhesion |
| Ednrb | Endothelin receptor type B, involved in vasoconstriction and development |
| Eci2 | Enoyl-CoA delta isomerase 2, involved in fatty acid metabolism |
| Cnga1 | Cyclic nucleotide-gated channel alpha 1, involved in visual signal transduction |
| Hk2 | Hexokinase 2, key enzyme in glycolysis |
| Pde6a | Phosphodiesterase 6A, involved in phototransduction in retinal rods |
| Camk2b | Calcium/calmodulin-dependent protein kinase II beta, involved in synaptic plasticity |
| Neurod4 | Neurogenic differentiation 4, involved in neuronal development |
| Cabp5 | Calcium binding protein 5, involved in calcium signaling in retinal cells |
